# Metachronous multicentric giant cell tumor of bone with pulmonary metastases: a case report with 20-year follow-up

**DOI:** 10.3389/fonc.2026.1794750

**Published:** 2026-05-07

**Authors:** Lei Zhao, Wei Wang, Guohui Liang, Zhicai Zhang, Jianxiang Liu, Binlong Zhong

**Affiliations:** Department of Orthopaedics, Union Hospital, Tongji Medical College, Huazhong University of Science and Technology, Wuhan, China

**Keywords:** giant cell tumor of bone, multicentric giant cell tumor of bone, pulmonary metastasis, denosumab, case report

## Abstract

Giant cell tumor of bone (GCTB) is typically a solitary lesion, with multicentric metachronous GCTB (MGCTB) accompanied by pulmonary metastasis being exceedingly rare and posing notable management challenges. A case of MGCTB with 20 years follow up at Union Hospital, Tongji Medical College, Huazhong University of Science and Technology is reported, and relevant literatures are reviewed. This report details a 38-year-old male with MGCTB and pulmonary metastases, followed for over 20 years since his initial treatment in 2005. Despite multiple surgical interventions, including curettage, resection, and eventual amputation, the disease progressed with new osteolytic lesions emerging years later. Systemic therapy with denosumab, initiated in 2017, achieved sustained disease stabilization. Following a treatment interruption during the COVID-19 pandemic, denosumab was resumed in 2021 and effectively controlled systemic lesions; however, a persistently progressing rib lesion necessitated wide en bloc resection in 2022. To date, the patient continues on denosumab with well-controlled disease and no evidence of progression, illustrating its critical role in achieving long-term control in this rare, aggressive GCTB variant. MGCTB is extremely rare. This case underscores the importance of continuous systemic therapy in managing MGCTB, even in the context of surgical interventions.

## Introduction

Giant cell tumor of bone (GCTB) is a locally aggressive, borderline primary bone tumor that predominantly affects young adults aged 20 to 40 years. It comprises 4-5% of all the primary bone tumors and 20% of benign bone tumors, with a predilection for the epiphyseal-metaphyseal region of long bones (1). The distal femur and proximal tibia are the most commonly involved sites, representing roughly 50–65% of cases (2). Radiographically, GCTB typically presents as an eccentric, expansile osteolytic lesion with cortical thinning and a characteristic “soap-bubble” appearance, often accompanied by pathological fracture in advanced stages.

The primary treatment for GCTB remains surgical, with joint-preserving procedures such as extended curettage combined with local adjuvant therapies being the standard approach (3). Nevertheless, local recurrence rates remain notably high, ranging from 20.5% to 53%, with malignant transformation occurring in approximately 10% of cases (2, 4, 5). Among these, about 1–4% may develop pulmonary metastases. In cases where complete resection is unattainable or would result in severe functional impairment, denosumab—a monoclonal antibody targeting the RANK ligand—has emerged as an effective systemic therapy to inhibit osteoclast-mediated bone destruction and control tumor progression (6, 7).

Multicentric giant cell tumor of bone (MGCTB), characterized by the presence of two or more radiographically and histologically distinct lesions, represents an exceedingly rare clinical entity, comprising fewer than 1% of all GCTB cases (8). The co-occurrence of MGCTB with synchronous or metachronous pulmonary metastases is even more uncommon, posing considerable diagnostic and therapeutic challenges due to its aggressive behavior and unpredictable clinical course. MGCTB is classified based on the timing of lesion appearance into synchronous and metachronous types. Synchronous multicentric GCTB refers to the presence of multiple lesions at the time of initial diagnosis or with an interval between the appearances of lesions of less than six months (9–12). In contrast, metachronous multicentric GCTB involves non-simultaneous occurrences of multiple lesions, with intervals exceeding six months. In metachronous cases, additional lesions typically emerge within two years, although some patients may experience intervals extending up to 20 years or longer (13). Although synchronous tumors are less common than metachronous tumors, the exact incidence of the two, the causes of the difference and the prognosis of the two are unknown.

Herein, we present an exceptional case of metachronous MGCTB with pulmonary metastasis in a patient managed over a 20-year period. Despite multiple prior surgical interventions, the patient ultimately achieved sustained radiographic and clinical stabilization through long-term denosumab therapy, underscoring its pivotal role in controlling advanced, multifocal disease. This case not only exemplifies the complexities involved in managing rare MGCTB variants but also highlights the potential of targeted biologic therapy to facilitate prolonged disease control in complex, metastatic presentations.

## Case report

**Figure 1:**
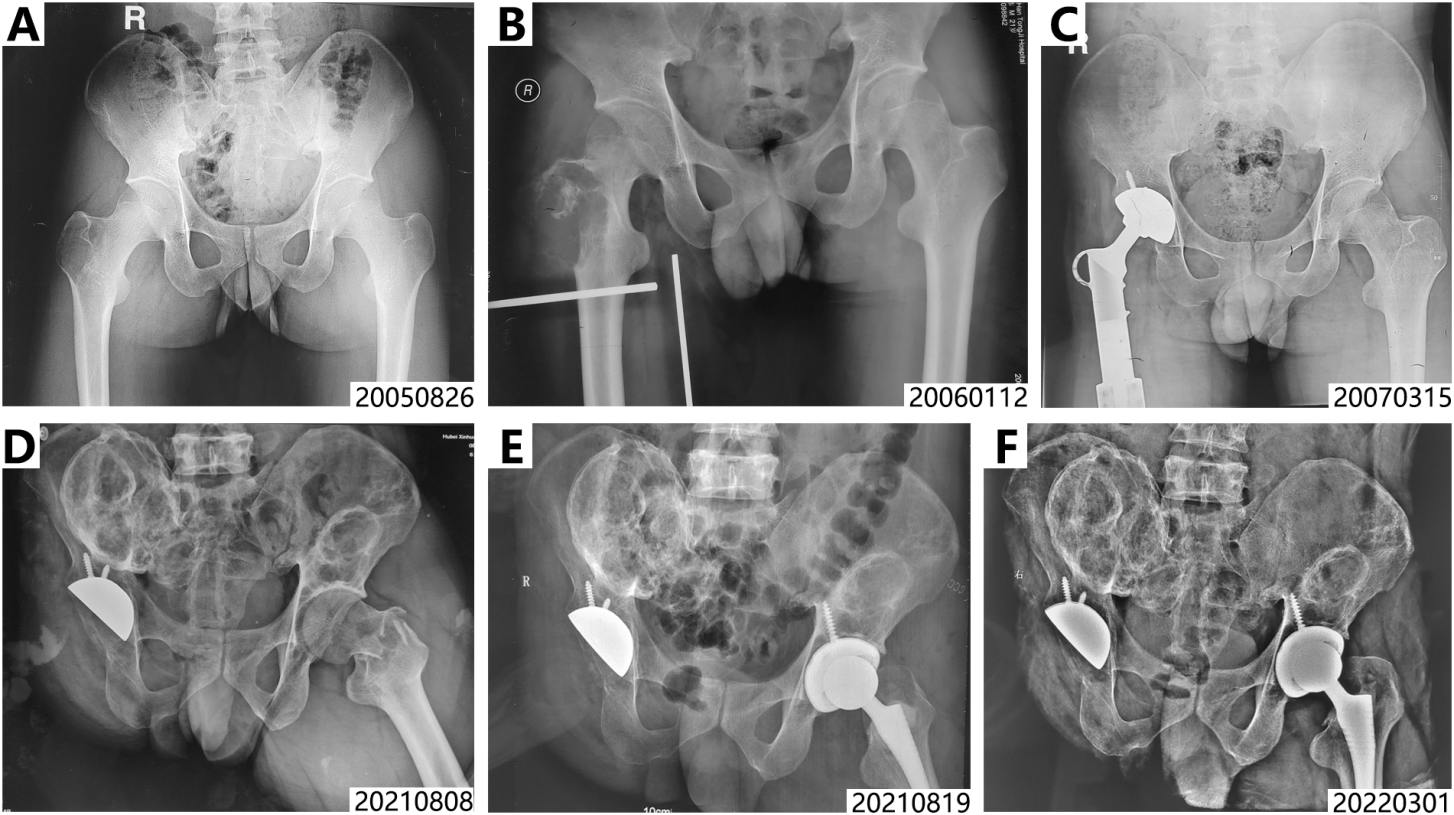
X rays of proximal femur and pelvic from Aug. 2005 to Mar. 2022. **(A)** The first osteolytic lesion discovered in greater trochanter of left femur in August 2005. **(B)** Tumor recurrence of left femur in January 2006. **(C)** X ray of proximal femur after right hip arthroplasty in March 2007, no osteolytic lesions were found in pelvic and left femur. **(D, E)** Pre- and post- hip arthroplasty X-rays from August 2021 document the management of a left intertrochanteric pathological fracture due to the discontinued therapy of denosumab. **(F)** Pelvic lesions manifest as more localized and circumscribed areas with increased calcification after denosumab resumption.

**Figure 2:**
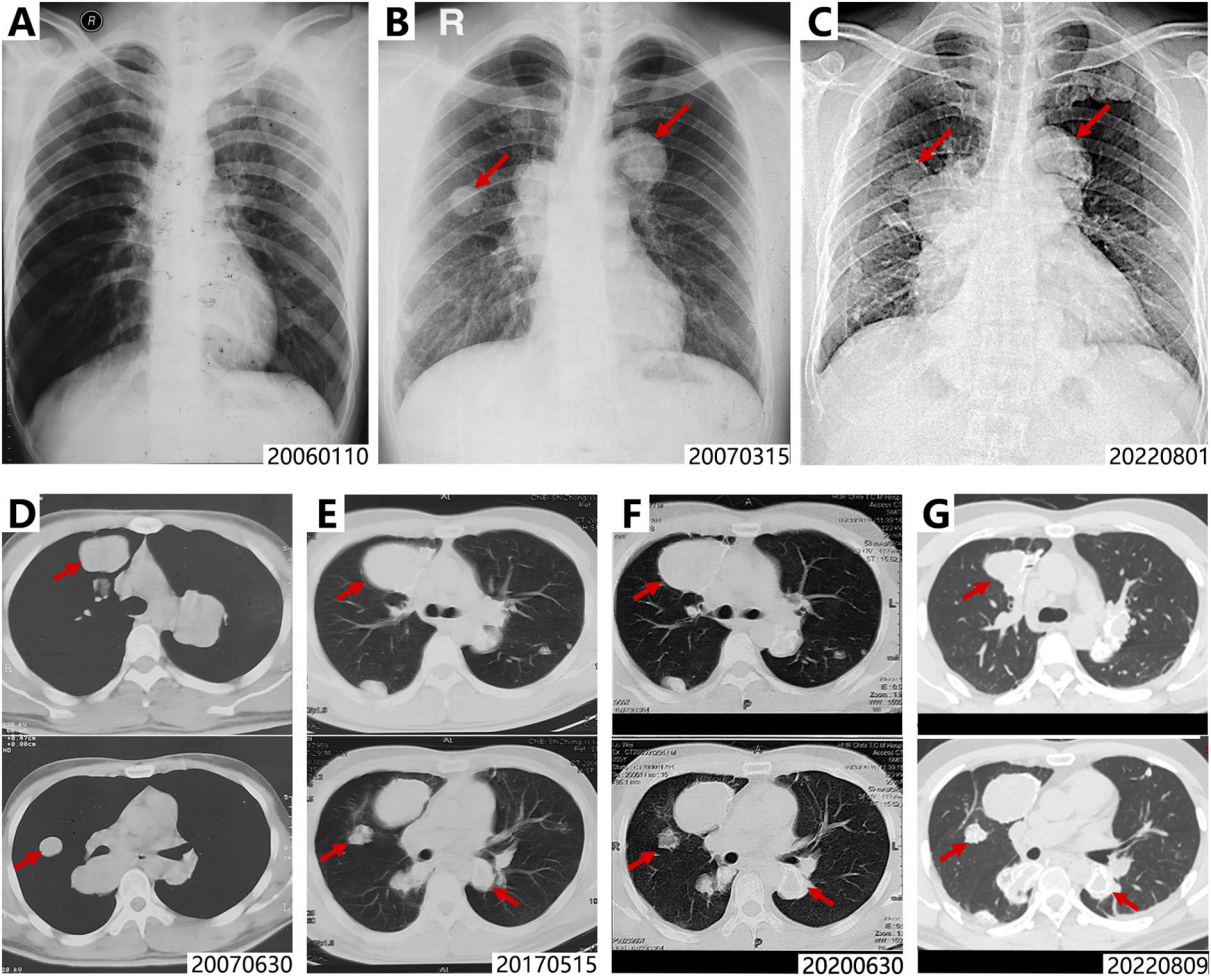
X ray and CT imaging of pulmonary metastases from Jan. 2006 to Aug. 2022. **(A–C)**. Chest radiographs show rapid progression of pulmonary metastases (2006-2007), with gradual control achieved following chemotherapy and denosumab therapy (2007-2022). **(D–G)** CT imaging documented progressive tumor growth from 2007 to 2017, which was followed by disease stabilization and the emergence of characteristic peripheral rim calcification upon initiation of denosumab therapy in 2017. Red arrows denote typical changes in partial lesions.

A 38-year-old male patient presented in 2022 with a chief complaint of a gradually enlarging mass in the right 10th rib over a 9-month period. His medical history dates back to August 2005, when he developed right hip pain. Radiographs at that time revealed an osteolytic lesion in the right greater trochanter (**Figure 1A**). The patient underwent curettage and bone grafting of the right proximal femoral tumor at an outside institution, with postoperative pathological examination confirming the diagnosis of GCTB. One month postoperatively, the tumor recurred subcutaneously and was excised again. In January 2006, the patient experienced recurrence of right hip pain, and follow-up imaging demonstrated osteolytic destruction in the right proximal femur along with newly detected pulmonary nodules (**Figure 1B**), suggestive of local tumor recurrence accompanied by pulmonary metastases. Subsequently, the patient underwent resection of the right proximal femoral tumor and total hip arthroplasty (**Figure 1C**). Pathological analysis confirmed GCTB (Jaffe grade III) without evidence of malignant transformation. Between 2006 and 2007, he received multiple cycles of systemic chemotherapy, including regimens of AP (doxorubicin + cisplatin) and IAP (ifosfamide + doxorubicin + cisplatin). Despite chemotherapy, follow-up imaging showed continued progression of the pulmonary metastatic lesions (**Figures 2A, B**).

**Figure 3:**
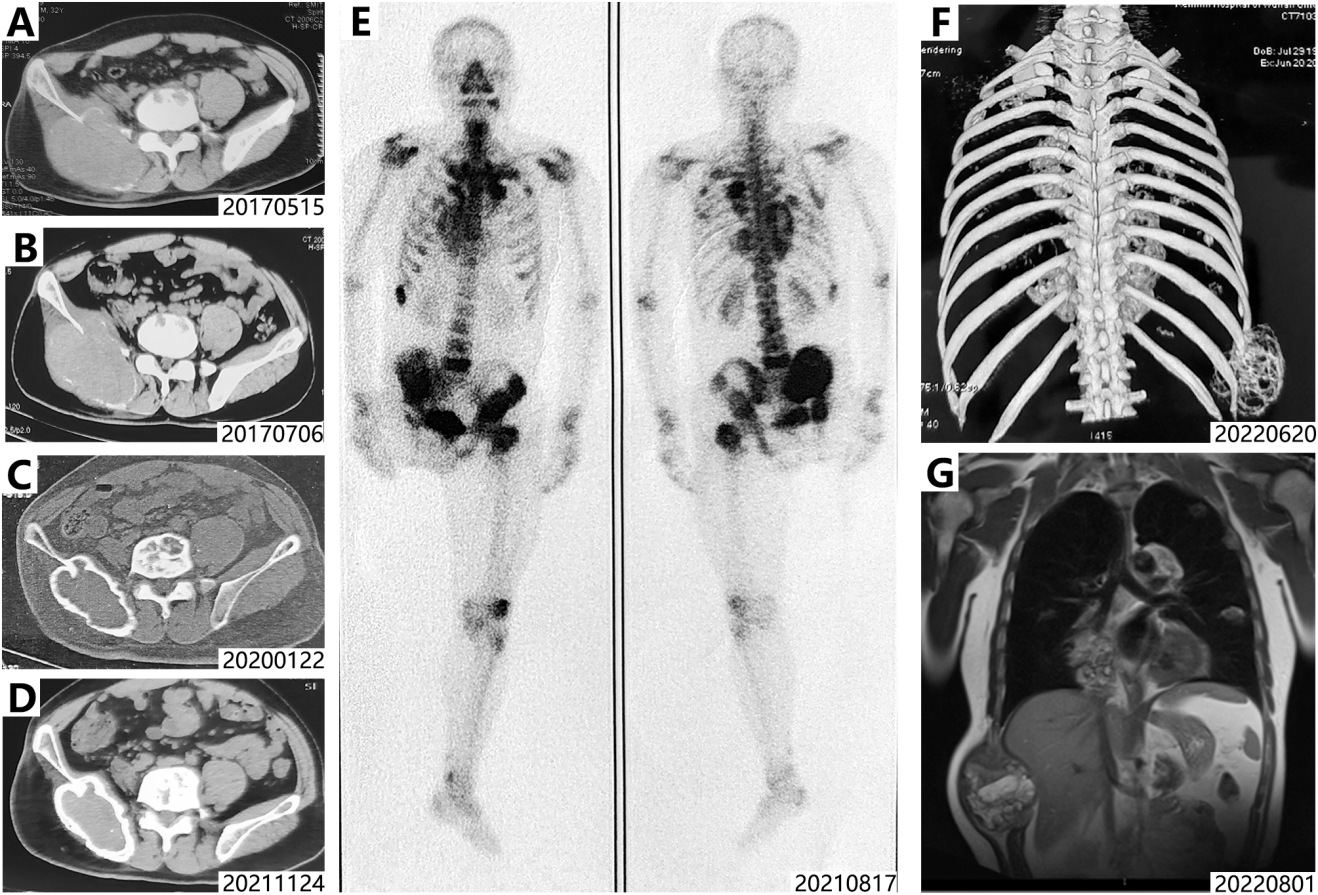
Imaging of pelvic and rib lesions and bone scintigraphy. **(A, B)** Slowed progression and fine peripheral rim calcification were found in pelvic lesions after initial denosumab therapy. **(C, D)** Reduction in size and development of distinct rim calcification were observed in pelvic lesions after denosumab treatment; no progression was noted on long-term follow-up. **(E)** Bone scintigraphy of August 2021 revealed multiple osteolytic lesions, consistent with multicentric giant cell tumor of bone. **(F)** CT scan of the ribs in June. 2022 reveals a large mass (approximately 9 cm × 8.5 cm × 10 cm) at the axillary segment of the right tenth rib. **(G)** MRI reveals a heterogeneous signal intensity lesion involving the right tenth rib, predominantly hyperintense on T2-weighted images. The lesion displaces the right lobe of the liver medially and protrudes outward from the chest wall.

In 2012, a progressively enlarging mass emerged at the original surgical site on the right thigh, indicating local tumor recurrence. Limb salvage was no longer considered feasible, and a right hip disarticulation was consequently performed. In 2017, a recurrent mass was noted in the right buttock, while imaging revealed metastatic involvement of the bilateral iliac bones, lumbosacral spine, and right kidney, alongside slight progression of the pulmonary metastases (**Figures 2**, **3A, B, E**). As a result, denosumab therapy was initiated in July 2017. Following treatment initiation, the pulmonary metastases gradually became controlled and calcified. Concurrently, the soft-tissue masses in the right ilium and right kidney demonstrated sclerosis and shrinkage (**Figures 1D–F**, **2D, E**, **3A–D**).

Unfortunately, due to disruptions caused by the COVID-19 pandemic, the patient was unable to secure denosumab through overseas channels and subsequently discontinued treatment from December 2019 to October 2021. In August 2021, following a fall, he sustained a pathological fracture at the base of the left femoral neck (**Figure 1D**). An ECT scan performed thereafter revealed multiple hypermetabolic foci in the lungs, thoracolumbar spine, ribs, pelvis, left femoral neck, and distal femur, consistent with widespread metastatic disease (**Figure 3E**). Ultimately, wide resection of the left femoral lesion and total hip arthroplasty were performed.

Following the identification of a mass in the right hypochondriac region, denosumab therapy was resumed in November 2021. Between January and June 2022, the lesion demonstrated progressive enlargement. CT and MRI revealed osteolytic destruction of the right 10th rib accompanied by a sizable soft-tissue mass, which caused displacement of the right hepatic lobe (**Figures 3F, G**).

Due to the progressive enlargement of the right 10th rib tumor and associated intermittent pain that impacted daily activities, the patient underwent *en*-bloc resection at our institution. Histopathological examination confirmed metastatic giant cell tumor of bone with a secondary aneurysmal bone cyst component and hemorrhage. Postoperatively, denosumab therapy was continued at a standard dosage of 120 mg monthly. No additional local interventions were administered during the subsequent follow-up. Over a 3-year postoperative follow-up period, there was no evidence of local recurrence at the right 10th rib site, and all remaining metastatic lesions remained radiologically stable without progression.

**Figure 4:**
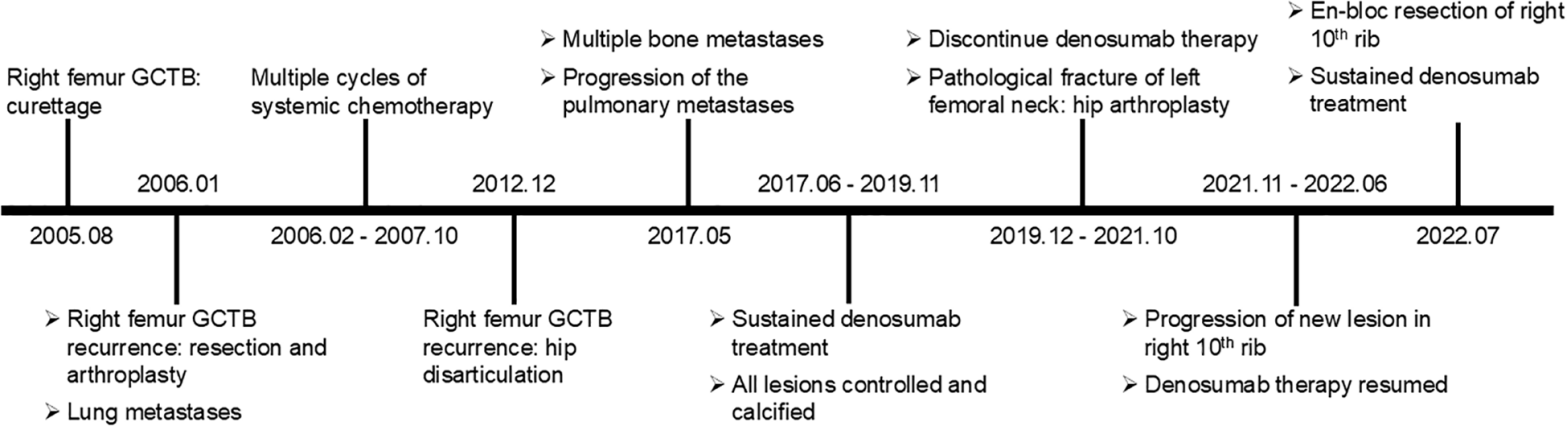
Clear and effective timeline of this case.

A concise summary of the diagnostic and therapeutic timeline is provided in **Figure 4**.

## Discussion

MGCTB is relatively rare and predominantly affects young adults around 20 years of age, with a higher incidence in females. The predilection sites are generally similar to those of solitary GCTB. The underlying pathological mechanisms of multicentric GCTB remain unclear. Proposed hypotheses include direct seeding to adjacent sites, iatrogenic dissemination of tumor cells, hematogenous spread, and malignant transformation of GCTB (13). While solitary benign GCTB has a known tendency for pulmonary metastasis and malignant change, the histocytological features of multicentric GCTB often resemble those of solitary benign lesions. This similarity suggests that multicentric GCTB may not arise from metastasis but rather represents primary tumors developing at multiple sites.

The primary goals in managing MGCTB are to control disease progression, eradicate lesions, and preserve limb function. For resectable lesions, intralesional curettage with cement filling remains the preferred initial approach, despite its high associated recurrence rate. Although wide resection is associated with a significantly lower recurrence rate (approximately 5%), it often necessitates joint arthroplasty, potentially leading to impaired joint function. Therefore, clinical decisions should carefully consider the patient’s overall condition and the specific characteristics of the lesions.

Histologically, GCTB consists of mononuclear spindle-shaped stromal cells expressing RANKL and multinucleated osteoclast-like giant cells expressing RANK. RANKL secreted by stromal cells recruits giant cells via the RANK-RANKL pathway, driving osteolytic destruction. Denosumab, a monoclonal antibody against RANKL, blocks this interaction, inhibits giant cell recruitment, halts osteolysis, promotes bone formation, and induces lesion calcification and stabilization. Prior to the US approval of denosumab in 2010, there were no established effective systemic therapies for patients with unresectable giant cell tumor of bone (GCTB), multicentric GCTB (MGCTB), or GCTB with pulmonary metastases. In this context, radiotherapy, chemotherapy, and interferon-alpha (IFN-α) were occasionally used as last resorts, representing the only potentially viable options despite the lack of validation from randomized clinical trials (14). For example, Sapiai et al. reported a patient with MGCTB who received chemotherapy; however, the disease continued to progress, ultimately leading to hemipelvectomy (11). At that time, whether such regimens conferred genuine clinical benefit or merely constituted overtreatment remained a matter of considerable debate (15, 16).

Since the introduction of denosumab, it has gradually become a cornerstone in the management of these challenging cases. Denosumab is now employed as neoadjuvant therapy prior to surgical intervention or as palliative treatment for unresectable and advanced GCTB. Extensive studies indicate that denosumab treatment can achieve satisfactory tumor control or downstaging in patients with unresectable GCTB. A recent multicenter phase II clinical trial involving 520 patients with unresectable GCTB treated with denosumab reported favorable disease control, with disease progression occurring in only two patients (<1%) (17). In the second cohort of this study (248 patients), 37% no longer required surgery, and 44% underwent less extensive procedures than originally planned. Among patients initially scheduled for radical surgeries such as total spondylectomy, hemipelvectomy, amputation, joint replacement, or arthrodesis, 96% were able to switch to less invasive surgical options, thereby preserving joint and neurological function.

For unresectable MGCTB, long-term treatment with RANKL inhibitors, such as denosumab, can be considered to control disease progression and alleviate or eliminate symptoms (13). While there are reported cases in which denosumab has been used as a non-surgical alternative for MGCTB, a clear consensus on its definitive efficacy and standardized treatment protocols remains to be established (8, 10). Lin et al. reported a patient with multicentric giant cell tumor of bone (MGCTB) who received 12 injections of denosumab. Postoperative histopathological examination revealed no residual tumor in the tibial lesion, thereby providing pathological evidence supporting the efficacy of denosumab in the treatment of MGCTB (8). Poudel et al. reported two cases of MGCTB managed with denosumab alone, achieving satisfactory symptom relief and tumor control, with extensive calcification observed across multiple lesions and no radiographic progression during a three-year follow-up (10). Similarly, Vaishya et al. described a case of MGCTB where denosumab was used post-surgical intervention to control multifocal progression. At one-year follow-up, symptoms had completely resolved, obviating the need for surgical resection of knee and pelvic lesions (18). Reports on denosumab use in MGCTB remain limited, with short follow-up periods, leaving the long-term efficacy of this approach still under investigation.

In the present case, the patient developed pulmonary metastases in 2006. At that time, as denosumab had not yet been approved for clinical use in China, the patient was only able to receive multiple cycles of chemotherapy. Despite this intervention, the lesions in the lungs, right proximal femur, and pelvis exhibited continuous progression, resulting in an unfavorable clinical course. In July 2017, the patient independently procured denosumab from overseas sources—given that the drug was not approved in mainland China until 2019—and subsequently initiated treatment. Following the commencement of denosumab therapy, the patient achieved significant radiographic calcification and sclerosis across multiple systemic lesions as well as the pulmonary metastases, accompanied by sustained disease stabilization and no evidence of further progression. This initially confirmed the effectiveness of denosumab in controlling the disease in this patient. However, treatment was interrupted in 2020 due to the COVID-19 pandemic, after which the tumor showed slow progression, eventually resulting in a pathological fracture of the left femur. This highlights the critical importance of continuous drug exposure for maintaining therapeutic efficacy. Following the detection of a new rib lesion in November 2021, treatment was restarted, but local control of this lesion did not meet expectations. This discrepancy may be explained by several factors. Denosumab targets RANKL on osteoclast-like giant cells but does not directly eliminate the proliferative mononuclear stromal cells—the true drivers of GCTB progression (19). Additionally, the rib lesion exhibited GCTB with concurrent ABC on postoperative pathology. The RANK-RANKL pathway is involved in ABC pathogenesis, but ABCs may respond heterogeneously to denosumab, and denosumab remains off-label for this indication (20, 21). Finally, clonal heterogeneity across lesions cannot be excluded, potentially conferring relative resistance in the rib subclone. These findings indicate that responses to denosumab can be lesion-specific, particularly in mixed GCTB/ABC lesions, emphasizing the need for individualized lesion monitoring.

This disease course evolution underscores several unresolved key issues in current clinical practice: First, the optimal timing for dose reduction or discontinuation of denosumab in patients who have achieved long-term disease stability remains undefined. Second, whether denosumab exerts consistent and sustained control over all lesions, particularly those in specific anatomical sites, requires further validation. Notably, the postoperative pathology of the resected rib lesion in this case revealed GCTB combined with an ABC. Whether this unique pathological composition is associated with the observed resistance to denosumab at this local site remains unclear.

In conclusion, MGCTBs represent an exceedingly rare clinical entity, posing significant management challenges. Denosumab may serve as a primary therapeutic option for disease control in these complex cases. Future multicenter, large-sample, long-term follow-up studies are imperative to more precisely evaluate the overall efficacy and limitations of denosumab in treating MGCTB, thereby providing high-level evidence for the development of individualized treatment strategies.

## Data Availability

The original contributions presented in the study are included in the article/supplementary material. Further inquiries can be directed to the corresponding author.
